# Portal hypertension in biliary atresia: a Japanese biliary atresia registry study

**DOI:** 10.1007/s00383-025-06222-4

**Published:** 2025-10-31

**Authors:** Ryuji Okubo, Masaki Nio, Hideyuki Sasaki, Taichi Fukuzawa, Hironori Kudo, Ryo Ando, Tsuyoshi Sakurai, Masatoshi Hashimoto, Keisuke Tada, Motoshi Wada, Japanese Biliary Atresia Society

**Affiliations:** 1 Department of Pediatric Surgery, Tohoku University Graduate School of Medicine, 1-1, Seiryou-machi, Aoba-ku, Sendai, Miyagi 980-8574 Japan; 2 Department of Surgery, Miyagi Children’s Hospital, 4-3-17, Ochiai, Aoba-ku, Sendai, Miyagi 989-3126 Japan; 3 Division of Pediatric Surgery, Department of Surgery II, Faculty of Medicine, Yamagata University, 2-2-2 Iidanishi, Yamagata, 990-9585 Japan; 4 The Japanese Biliary Atresia Society, The Office of the Japanese Biliary Atresia Society, 1-1, Seiryou-machi, Aoba-ku, Sendai, Miyagi 980-8574 Japan

**Keywords:** Biliary atresia, Portal hypertension, Gastroesophageal varices, Hypersplenism, Long-term follow-up

## Abstract

**Purpose:**

This study aimed to clarify the incidence of portal hypertension (PH) in biliary atresia (BA), its impact on long-term native liver survival rates (NLSRs), and the role of PH treatment.

**Methods:**

Data were analyzed from 3,777 patients in the Japanese Biliary Atresia Registry (1989–2021). Incidence and treatment of PH were examined in all patients and in 20-year native liver survivors. A subgroup of 596 jaundice-free native liver survivors was divided into three groups: without PH (A), untreated PH (B), and treated PH (C). Patient characteristics, clinical outcomes, and survival analyses were performed.

**Results:**

PH occurred in 46.4% of all patients, with gastroesophageal varices (35.1%) and hypersplenism (36.8%) being most common. Among 759 20-year native liver survivors, 49.8% had PH, largely diagnosed between 2 and 15 years. Group C patients were older at Kasai portoenterostomy and had more cholangitis. Survival analysis showed best outcomes in group A and worst in group C. Conditional native liver survival analysis revealed group B initially had better NLSRs than group C, but differences diminished beyond 15 years.

**Conclusion:**

In native liver survivors without jaundice following KP, PH influenced prognosis, but successful treatment enabled long-term survival comparable to patients with mild or no PH.

## Introduction

Biliary atresia (BA) is a rare disease involving the entire liver and biliary system, which develops during the neonatal period or early infancy, with an incidence of 1 in 8,000–15,000 live births. However, surgery is required to ensure patient survival. Kasai portoenterostomy (KP) is the first-line surgical modality [1, 2] and liver transplantation (LTx) is necessary in 50% of patients before adulthood [3, 4].

Although the main reason for LTx is liver failure due to persistent or recurrent jaundice after KP, the development of severe portal hypertension (PH) can also be a reason for LTx.

The incidence of PH after KP is between 24 and 96% [5–8]. PH can manifest with various symptoms, such as gastroesophageal varices (GEV) [5–16], hypersplenism (HS) [11, 17], gastrointestinal bleeding (excluding varices) (GIB) [18, 19], hepatopulmonary syndrome (HPS) [17, 20–22] and portopulmonary hypertension (PoPH) [23]. Depending on the severity of symptoms, the use of medical and/or surgical therapeutic modalities for PH is considered [9, 10, 16, 24–27]. Furthermore, patients with intractable PH might be candidates for LTx. Although PH is a serious problem in patients with BA following KP, long-term outcomes have not been well documented.

The Japanese Biliary Atresia Registry (JBAR) was launched in 1989 by the Japanese Biliary Atresia Society (JBAS) to investigate the epidemiology and etiology of BA and improve surgical outcomes [28, 29]. As of 2021, 3,777 patients have been registered. In Japan, deceased donor transplantation remains uncommon compared with Western countries, and the country relies heavily on living donor transplantation. Under these unique circumstances, the analysis of a globally unprecedented, large-scale, long-term data on BA represents a highly significant achievement.

In this study, we investigated the incidence of PH and impact of PH on native liver survival rates (NLSRs) in patients with BA and evaluated the role of PH treatment using JBAR data.

## Methods

### Patients

In the JBAR registry, an initial questionnaire was administered annually and follow-up questionnaire was scheduled at the 2nd, 5th, 10th, 15th, 20th, 25th, 30th, 35th, and 40th year after KP or primary LTx. Patients who underwent LTx were required to be enrolled in LTx registration. Regarding the occurrence and treatment of PH at each follow-up, new events from the previous follow-up were registered. Between 1989 and 2021, 3,777 patients were registered in the JBAR, and there were 759 20-year native liver survivors.

### Frequency of PH

Using JBAR data, we examined the frequencies of PH: GEV, HS, GIB, HPS, and PoPH. A diagnosis of HS was based on thrombocytopenia and the presence of splenomegaly together with complications, typically bleeding. GIB included portal hypertensive gastropathy, portal hypertensive enteropathy, portal hypertensive colonopathy, and ectopic varices. HPS was defined as a disorder of gas exchange caused by intrapulmonary shunting secondary to liver disease–associated intrapulmonary vascular dilation. PoPH was defined as a mean pulmonary arterial pressure ≥ 25 mmHg at rest, a pulmonary wedge pressure ≤ 15 mmHg, and a pulmonary vascular resistance ≥ 240 dyn·s·cm⁻⁵. Although PH is formally defined as a hepatic venous pressure gradient of > 5 mmHg, in this study, where such measurements were not available for all patients, it was defined by the presence of representative clinical manifestations of PH [30]. Additionally, we examined the presence of treatment for GEV and HS. The total number and proportion of patients who experienced each symptom or required treatment at least once during the follow-up period were also calculated. As the symptoms and findings of PH can improve with treatment or over the natural course, we conducted a similar study among the 20-year native liver survivors.

### Timing of PH onset and treatment in native liver survivors

Timing of the onset of GEV, HS, HPS, and PoPH was examined at follow-up registration in native liver survivors. We also examined timing of treatments for GEV and HS.

### Impacts of the presence and severity of PH on prognosis

**Fig. 1:**
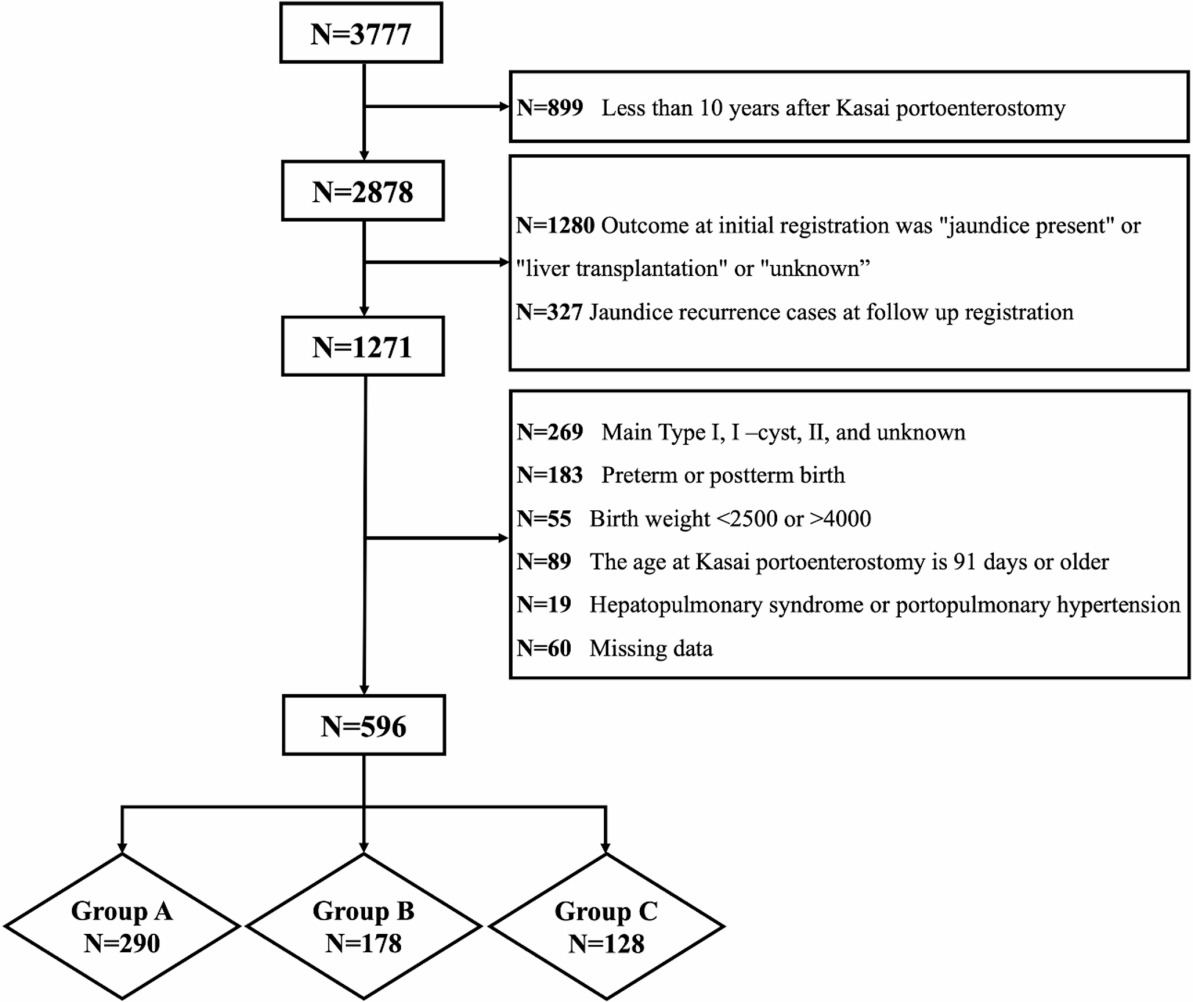
Flow chart for patients. Type I: atresia of the common bile duct. Type I cyst: atresia of the common bile duct with a cystic structure that communicates with the intrahepatic bile ducts. Type II: atresia of the hepatic duct Of the 3,777 patients, 596 were included in the analysis of the impact of the presence and severity of portal hypertension on prognosis and were divided into three groups, A, B, and C, for comparison

To examine the characteristics of PH and its impact on prognosis, we selected patients based on the following inclusion criteria: native liver survivors aged ≥ 10 years without jaundice, the main type III: biliary obstruction at the porta hepatis [31], age at KP < 90 days, full-term infants with normal birth weight at delivery, and no association of HPS or PoPH. Patients with persistent or recurrent jaundice after KP were excluded because jaundice is an independent prognostic factor. Obstruction type and age at KP were also significant prognostic factors following KP [32, 33]. Additionally, birth weight and gestational age were included as criteria because they were thought to affect postoperative outcomes, such as the difficulty of surgery due to immaturity and small physique. Patients with HPS and PoPH were excluded from further analysis because these conditions were indications for Ltx. A total of 596 patients were selected (Fig. 1). We classified the patients into three groups: group A, without PH; group B, with untreated PH; and group C, with treated PH. PH treatment included endoscopic and surgical treatment of GEV and splenectomy or partial splenic embolization for HS.

In 596 patients, we examined sex, gestational age, birth weight, associated anomalies, total bilirubin (T-Bil) and direct bilirubin (D-Bil) levels at KP, age at KP, reoperation, and cholangitis within 1 year postoperatively. Associated anomalies included polysplenia, asplenia, preduodenal portal vein, absence of the inferior vena cava, malrotation, situs inversus, and congenital heart diseases. Jaundice clearance was defined as T-Bil levels within normal range according to each hospital’s laboratory criteria, which was assessed after KP. Cholangitis was defined as fever and inflammatory findings, along with elevated levels of T-Bil and biliary enzymes. NLSRs were analyzed and compared between the three groups.

### Conditional native liver survival analysis in patients with PH

We compared the conditional native-liver survival rates between groups B and C to investigate the effects of PH treatment on every 5-year native liver survival period.

### Ethical approval

This study protocol was approved by the Clinical Research Ethics Board of the Tohoku University Graduate School of Medicine (2025-1-283).

### Statistical analyses

Descriptive data were summarized as numbers (percentages) for categorical variables and medians (interquartile ranges) for continuous variables. Statistical comparisons among the three groups were performed using the Kruskal–Wallis test for three unpaired continuous variables and the Pearson chi-square test for dichotomous variables. The Bonferroni test was used as a post-hoc test, and a *p* value of < 0.0167 was considered statistically significant among the three groups. Survival rates were analyzed using the Kaplan–Meier method, and the log-rank test was used to compare long-term survival distributions between the groups. Statistical analyses were performed using SPSS 16.0.0 (SAS Institute Japan Ltd., Tokyo, Japan).

## Results

### Frequency of PH

Of the 3,777 participants, 46.4% (1,753) had PH, 35.1% (1,326) had GEV, 36.8% (1,390) had HS, 4.7% (179) had GIB, 1.9% (71) had HPS, and 0.7% (27) had PoPH. There were 14.5% (549) of the patients with a history of treatment for GEV and 5.9% (224) with a history of treatment for HS (Table 1). Similarly, among the 20-year native liver survivors, the frequency of PH, GEV, HS, GIB, HPS, and PoPH were 49.8% (378), 43.2% (328), 39.3% (298), 5% (38), 0.1% (4), and 0.1% (7), respectively.

**Table 1:** Portal hypertension

	All registrants (*N* = 3,777)		20-year native liver survivors (*N* = 759)	
	N	(%)	N	(%)
Portal hypertension	1753	(46.4)	378	(49.8)
Gastroesophageal varices	1326	(35.1)	328	(43.2)
Treatment of gastroesophageal varices	549	(14.5)	104	(13.7)
Hypersplenism	1390	(36.8)	298	(39.3)
Treatment of hypersplenism	224	(5.9)	80	(10.5)
Gastrointestinal bleeding (excluding varices)	179	(4.7)	38	(5.0)
Hepatopulmonary syndrome	71	(1.9)	4	(0.1)
Portopulmonary hypertension	27	(0.7)	7	(0.1)
(Includes duplications)				

### Timing of PH onset and treatment in native liver survivors

**Fig. 2:**
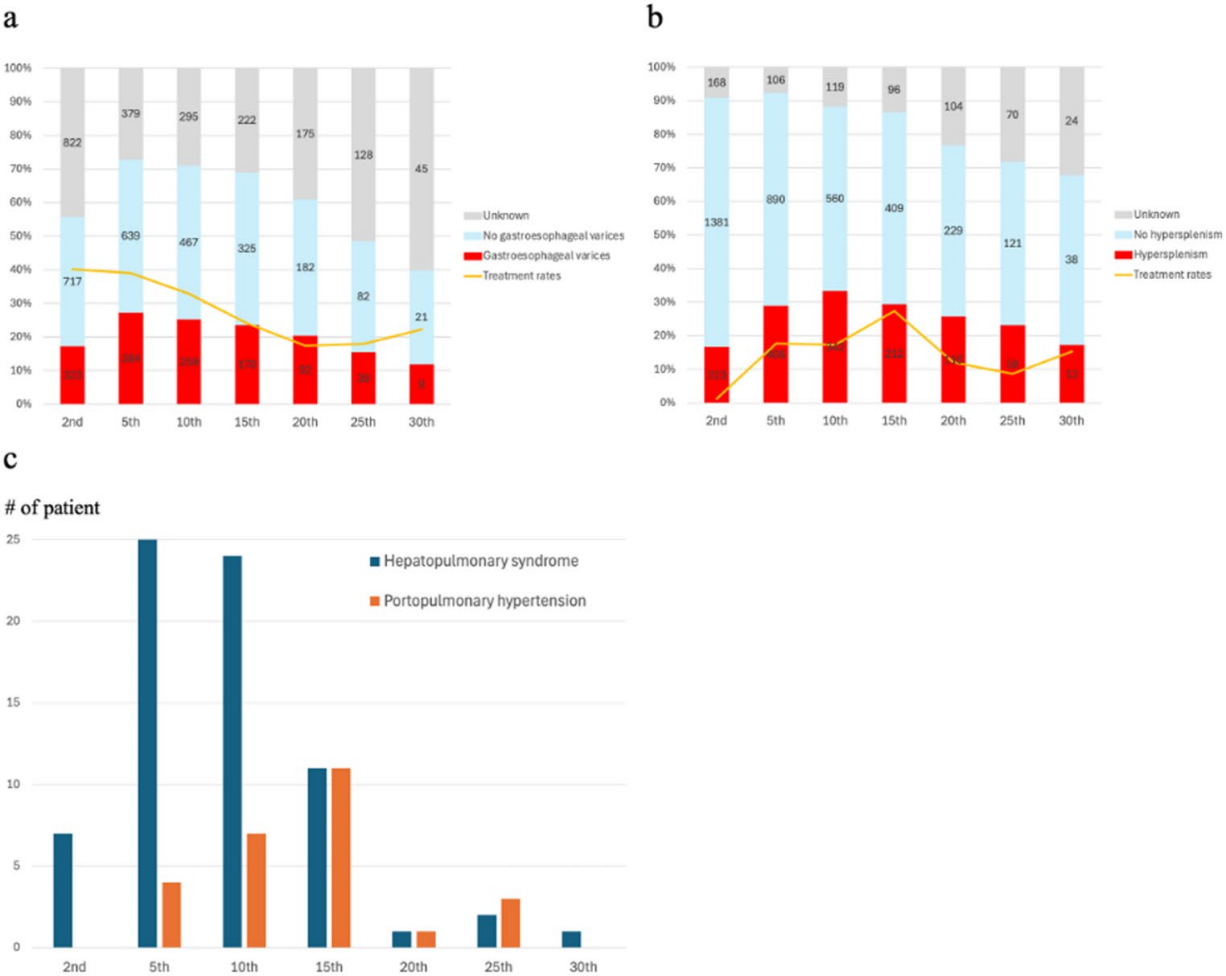
Timing of Portal hypertension onset and treatment in native liver survivors. Timing of PH onset and treatment: Gastroesophageal varices (Fig. 2a), hypersplenism (Fig. 2b), and hepatopulmonary syndrome and portopulmonary hypertension (Fig. 2c). The incidences of gastroesophageal varices, hypersplenism, hepatopulmonary syndrome, and portopulmonary hypertension were peaked at the 5th, 10th, 5th, and 15th, respectively. Treatment for gastroesophageal varices began in early childhood, and that for hypersplenism peaked at 15 years of age

The incidence of GEV peaked at 5 years of follow-up, and HS peaked at 10 years of follow-up. The incidence of HPS began at 2 years of follow-up, was most prevalent between 5 and 10 years of follow-up and then decreased. PoPH was absent at 2 years of follow-up, first detected at 5 years of follow-up, and peaked at 15 years of follow-up (Fig. 2). Treatment for GEV began in early childhood, and that for HS peaked at 15 years of follow-up. The onset and treatment rates showed downward trends after adolescence.

### Patient characteristics and clinical outcomes in 596 patients

Incidence rates of GEV in groups B and C were 71.9% and 97.7%, respectively. The incidences of HS in groups B and C were 69.1% and 88.3%, respectively. In group C, the age at KP was later than that in the other groups (*p* = 0.0003). The frequency of cholangitis was also significantly higher in group C than that in the other groups (*p* = 0.0002). There were no significant differences among the three groups in terms of sex, gestational age, birth weight, associated anomalies, preoperative T-Bil and D-Bil levels, or reoperation rates (Table 2).

**Table 2:** Patient characteristics and clinical outcomes

	Group A *N* = 290	Group B *N* = 178	Group C *N* = 128	*p* value
Gastroesophageal varices, n (%)		128 (71.9)	125 (97.7)	
Hypersplenism, n (%)		123 (69.1)	113 (88.3)	
Female, n (%)	168 (57.9)	110 (61.8)	86 (67.2)	NS
Gestational age (weeks), Median [IQR]	39 [38, 40]	39 [38, 40]	39 [38, 40]	NS
Birth weight (g), Median [IQR]	3005 [2773, 3299]	2996 [2780, 3296]	2990 [2801, 3196]	NS
Associated anomaly, n (%)	26 (9.0)	23 (12.9)	15 (11.7)	NS
Total bilirubin (mg/dL), Median [IQR]	8.7 [7.2, 10.9]	8.6 [7.4, 10.8]	9.1 [7.6, 11.2]	NS
Direct bilirubin (mg/dL), Median [IQR]	5.8 [4.6, 7.3]	5.9 [4.8, 7.7]	6.4 [4.9, 7.8]	NS
Age at surgery (days), Median [IQR]	56 [43, 67] ^*1, *2^	62 [49, 72] ^*1^	63 [46, 76] ^*2^	0.0003
Reoperation, n (%)	9 (3.1)	13 (7.3)	9 (7.0)	NS
Cholangitis, n (%)	100 (35.5) ^*3, *4^	86 (50.9) ^*3^	68 (54.8) ^*4^	0.0002

Notes: ^*1^:*p* = 0.0017, ^*2^:*p* = 0.0007, ^*3^:*p* = 0.0015, ^*4^:*p* = 0.0003. A probability (*p*) value < 0.0167 was considered statistically significant between three groups

### Survival analysis depending on the presence of PH and its treatment

**Fig. 3:**
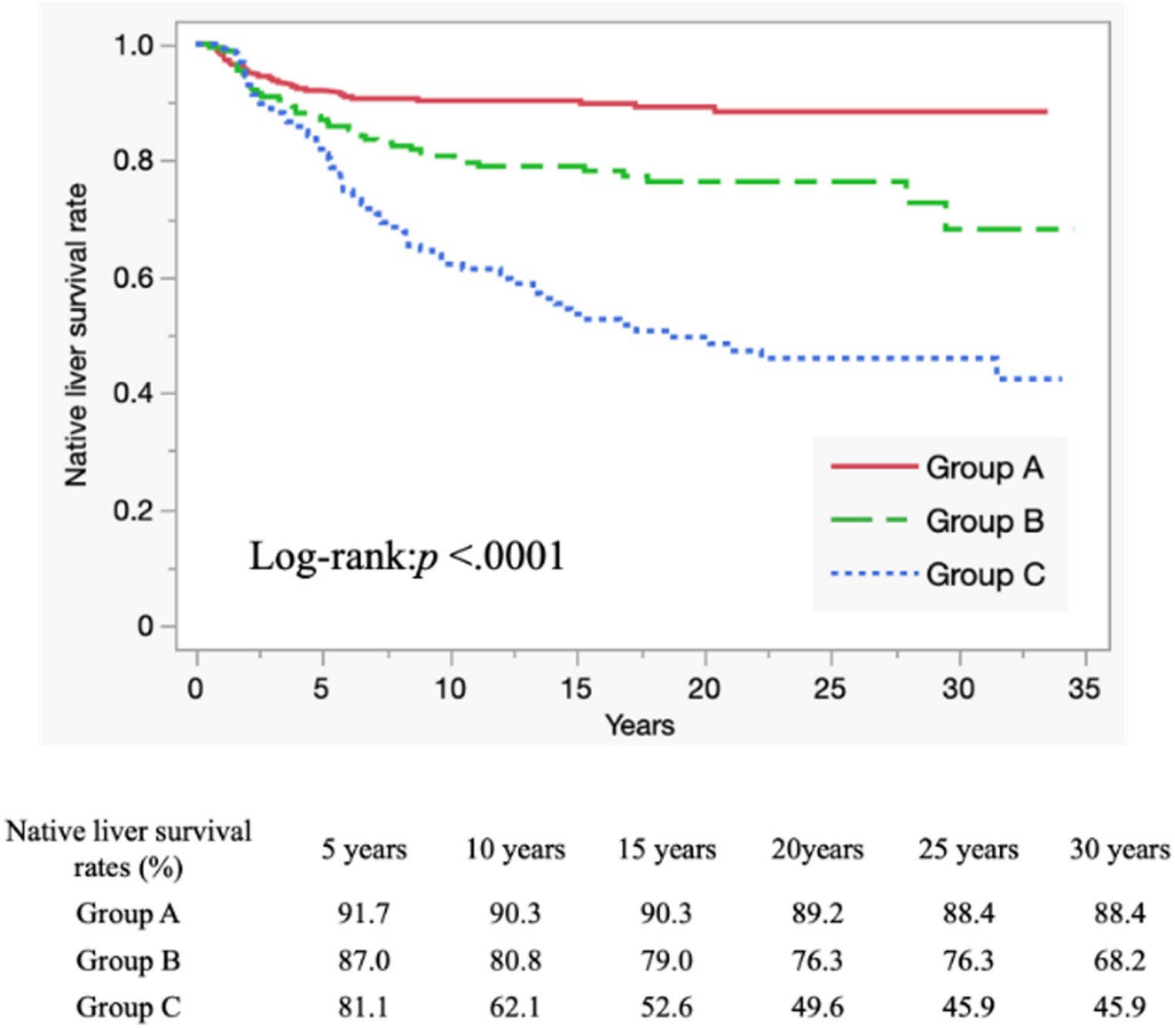
Kaplan–Meier survival curves and Native liver survival rates between the three groups. Native liver survival rates were the highest in group A and lowest in group C (log-rank test, *p* < 0.0001). The 10-, 20-, and 30-year native liver survival rates for each group were 90.3%, 89.2%, and 88.4% in group A; 80.8%, 76.3%, and 68.2% in group B; and 62.1%, 49.6%, and 45.9% in group C, respectively

NLSRs were highest in group A and lowest in group C (log-rank test, *p* < 0.0001). The 10-, 20-, and 30-year NLSRs for each group were 90.3%, 89.2%, and 88.4% in group A, 80.8%, 76.3%, and 68.2% in group B, and 62.1%, 49.6%, and 45.9% in group C, respectively (Fig. 3).

### Conditional native-liver survival analysis in patients with PH

**Fig. 4:**
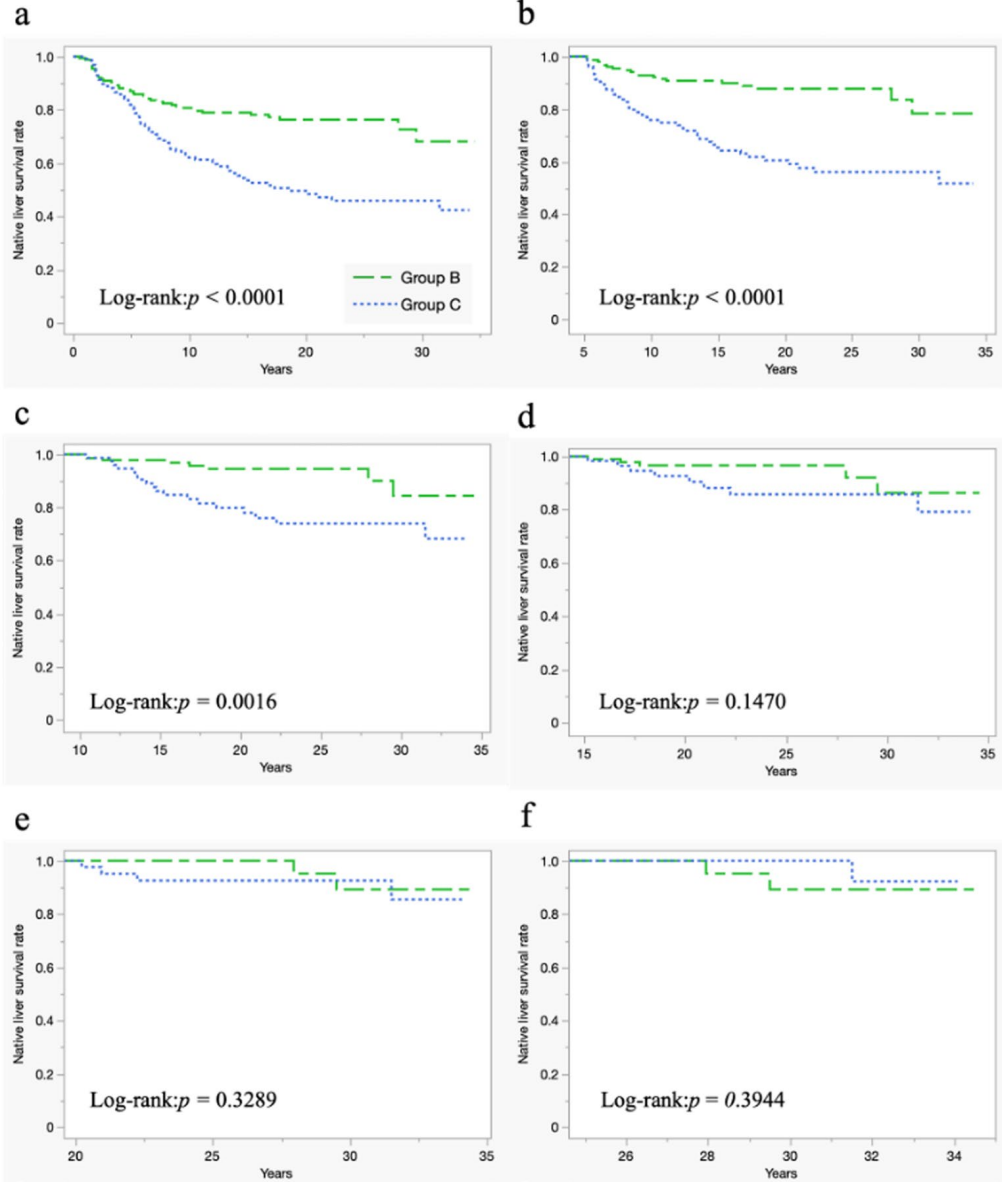
Conditional native-liver survival analysis in patients with Portal hypertension. Conditional Native Liver Survival Analysis: All cases (Fig. 4a), 5-year (Fig. 4b), 10-year (Fig. 4c), 15-year (Fig. 4d), 20-year (Fig. 4e), and 25-year (Fig. 4f) were native liver survivors. Native liver survival rates were significantly lower in group C than in group B for all cases and 5-year and 10-year native liver survivor. No significant differences were observed in 15-year, 20-year, and 25-year native liver survivors

In a conditional native liver survival analysis, NLSRs were significantly lower in group C than those in group B for all the patients (*p* < 0.0001), 5-year (*p* < 0.0001), and 10-year native liver survivors (*p* = 0.0018). However, no significant differences were observed in the 15-year (*p* = 0.1467), 20-year (*p* = 0.3209), and 25-year native liver survivors (*p* = 0.4123) (Fig. 4).

## Discussion

In the 1950s, the KP, a surgical treatment for BA, was developed, and its outcomes improved over time [34]. Furthermore, the widespread use of LTx since the 1980s has significantly enhanced the prognosis of BA [35]. As the number of long-term native liver survivors increases, various complications have become more evident. Among the major complications of BA, PH is one of the most important. Although there have been several reports on the incidence of these pathological conditions in patients with PH, there have been few studies on the long-term outcomes in native liver survivors without jaundice.

In this study, the prevalence of PH among all the JBAR registrants was 46.4% and 49.8% among the 20-year native liver survivors, indicating that nearly half of patients with BA had PH. These results are comparable to those of previous studies [5–8]. Although the prevalence of GEV and HS was similar, the number of patients who underwent treatment for GEV was approximately twice that of patients treated for HS. The incidence rates of GIB, HPS and PoPH were much lower than those of HS and GEV. Compared with the overall frequency, the incidence of PH in the 20-year native liver survivors was similar, but the incidences of HPS and PoPH significantly decreased.

The timing of the onset and treatment of each condition was also noteworthy. The GEV and HS were most frequently observed between the 2nd and 15th year after KP, after which gradually decreased. Treatment for GEV was initiated early, whereas HS treatment became more common after the 5 years of follow-up. This earlier intervention for GEV was likely due to the potentially life-threatening nature of GEV bleeding, prompting earlier treatment compared with HS. Additionally, splenectomy, one of the treatments for HS, was thought to be one of the reasons why the treatment was not performed during early childhood because of postoperative immune problems. HPS and PoPH showed a sudden increase around 5 years of follow-up, followed by a decline after 20 years of follow-up. Most patients with HPS and PoPH underwent LTx before the age of 20 years, which might explain the decline in the incidence of HPS and PoPH in adult patients. The onset of PH was not uniform; it tended to cluster, particularly during childhood and adolescence. Additionally, the timing of treatment varied, depending on the type of PH. Therefore, during postoperative follow-up, it is important to tailor examinations and treatments with an awareness of the timing and characteristics of each condition.

In cases where jaundice resolved after KP, significant differences were observed in the age at KP between patients with and without PH. Previous studies have reported that age at surgery was associated with jaundice clearance rates and NLSRs following KP [33, 36–38]. This study also suggested that the age at surgery might influence the development of PH. One possible explanation for the lower incidence of PH in patients who underwent surgery at an earlier age is that timely intervention might help mitigate the progression of liver damage. Furthermore, the higher frequency of cholangitis observed in patients with PH suggests that the inflammation caused by cholangitis might exacerbate liver fibrosis, potentially leading to the development of PH. Previous reports showed that age at KP and cholangitis were not related to the onset of PH [39], while others showed that cholangitis was related to PH [40]. This study was limited in scope to factors such as the number of years since KP and the jaundice recurrence, which may have affected the results. Even in patients whose jaundice resolves, careful monitoring of PH remains important, particularly in patients following late KP or those who experience postoperative cholangitis.

In the survival analysis, patients with PH showed a significantly greater decline in the NLSRs than those without PH. This study focused on patients who did not develop jaundice recurrence during the follow-up period. Nevertheless, even in such patients, the presence of PH, particularly PH requiring treatment, was found to have a significant negative impact on prognosis. Previous reports have also stated that GEV and HS in adolescents were poor prognostic factors [41, 42], and that patients with PH who require treatment should be prepared for LTx [43]. However, in the conditional native liver survival analysis, the impact of PH severity on prognosis changed over time. Among native liver survivors beyond 15 years postoperatively, the presence or absence of PH treatment no longer significantly influenced NLSRs. This suggests that the impact of PH severity on prognosis diminished over time. This implies that even if patients with PH requiring treatment during childhood or adolescence, long-term outcomes comparable to those of patients with milder or no PH may be achievable following appropriate management. One possible explanation for this improvement is the natural development of portosystemic shunts over time during prolonged PH, which can help alleviate PH severity. However, such shunting, in which portal blood bypasses the liver, can lead to complications, including HPS, PoPH, hepatic tumors, hyperammonemia, hypermanganesemia, and hepatic encephalopathy [44]. Therefore, careful long-term monitoring is essential for detecting and managing the potential adverse effects of portosystemic shunting, using methods such as oxygen saturation measurement, echocardiography, contrast-enhanced computed tomography, and blood test.

Another important consideration is that repeated treatments for PH may delay the appropriate timing of LTx. Furthermore, procedures such as splenectomy and partial splenic embolization may result in significant perisplenic adhesions, which can increase the technical difficulty of subsequent LTx. Additionally, as the duration of native liver survival, there is concern about the progression of cirrhosis due to worsening fibrosis. Therefore, regular assessment of hepatic functional reserve using blood tests, computed tomography, and ^99m^Tc-DTPA galactosyl human serum albumin liver scintigraphy is essential [45].

This study has several limitations. First, this was a retrospective study. Second, the follow-up period varied among the patients, and some patients were lost to follow-up, potentially leading to an underestimation of PH incidence and treatment frequency. Third, as data were collected from a multi-institutional database involving > 100 institutions, the diagnostic and treatment criteria for PH were not standardized, and practices may have differed across centers. Furthermore, the dataset spans more than 30 years, during which the management of PH and the indications for LTx in PH cases have evolved, potentially exerting a significant influence on the outcomes of this study. Furthermore, given the unique circumstances in Japan—such as the shortage of living donors, and the limited number of deceased donor transplantation—it cannot be denied that treatment for PH was prioritized even in cases where LTx would have been appropriate, potentially influencing the study results. Prospective studies focusing on PH cases are needed to clarify the effect of PH treatment on long-term prognosis of BA.

## Conclusions

In native liver survivors without jaundice following KP, PH affected the long-term prognosis. However, if patients with severe PH were successfully treated, native liver survival into adolescence and beyond was expected to be equivalent to that of patients with mild or no PH.

## Data Availability

Only the members of the Japanese Biliary Atresia Society can access to the data collected during this analysis when a proposal has been approved by the investigators and the Japanese Biliary Atresia Society. This rule was instituted by the bylaw of the Japanese Biliary Atresia Society.

## Abbreviations

BA: Biliary atresia
D-Bil: Direct bilirubin
GEV: Gastroesophageal varices
GIB: Gastrointestinal bleeding
HPS: Hepatopulmonary syndrome
HS: Hypersplenism
JBAR: Japanese biliary atresia registry
JBAS: Japanese biliary atresia society
KP: Kasai portoenterostomy
LTx: Liver transplantation
NLSRs: Native liver survival rates
PH: Portal hypertension
PoPH: Portopulmonary hypertension
T-Bil: Total bilirubin
